# IUC24431-79 Prognostic role of luminal and basal subtypes in metastatic prostate cancer: a retrospective study

**DOI:** 10.1093/oncolo/oyaf248.017

**Published:** 2025-08-29

**Authors:** S Merler, A Sordo, M Brunelli, F Zacchi, E Tasselli, L Tondulli, A Zivi

**Affiliations:** Oncology Department, San Maurizio Central Hospital of Bolzano, South Tyrolean Health Service, Italy; Centro Ricerche Cliniche, AOUI, Verona; Unit of Biostatistics, Epidemiology and Public Health, Department of Cardiac, Thoracic, Vascular Sciences and Public Health, University of Padua, Italy; Department of Diagnostics and Public Health, Pathology Unit, University and Hospital Trust of Verona, Italy; Centro Ricerche Cliniche, AOUI, Verona; Section of Innovation Biomedicine-Oncology Area, Department of Engineering for Innovation Medicine DIMI, University of Verona, Italy; Azienda Ospedaliera, Universitaria Integrata di Verona, Italy; Oncology Department, San Maurizio Central Hospital of Bolzano, South Tyrolean Health Service, Italy; Azienda Ospedaliera, Universitaria Integrata di Verona; Department of Engineering for Innovation Medicine DIMI, University of Verona, Italy

## Abstract

**Background:**

Metastatic hormone-sensitive prostate cancer (mHSPC) patients exhibit a wide spectrum of clinical outcomes, ranging from poor prognosis to long-term survival. Despite recent therapeutic advancements, treatment strategies for mHSPC are primarily based on clinical features (eg, high vs low volume disease) and molecular characteristics of the disease remain underexplored. The stratification between luminal vs basal disease subtypes is prognostic and predictive in breast cancer, and there is some evidence in metastatic castration-resistant prostate cancer (mCRPC) too. Thus, we aimed to explore such potential implications in mHSPC.

**Methods:**

Single-institution retrospective study of patients diagnosed with mHSPC and treated at the University Hospital of Verona from 2019. Tumor samples from diagnostic prostate biopsies were analyzed by immunoprofiling (IHC) using CK18, NKX3.1, AR, MYC, RB1, and ERG to define luminal and basal tumors. Clinical and pathological data were retrospectively collected. The primary endpoint of the study was overall survival (OS) within the 2 groups (luminal vs basal).

**Results:**

A total of 66 patients have been included, with a median follow-up of 38.5 months. Among them, 53 (80%) were classified as having luminal disease and 13 (19%) as basal. Clinical features and known prognostic factors were quite balanced between the 2 groups, with high-volume disease observed in 76% of basal and 62% of luminal cases, and de novo presentation in 84% and 83%, respectively. Notably, the luminal group showed a trend toward improved median OS compared to the basal group (not reached vs 47 months; HR 0.45, 95% CI, 0.18-1.10; *P* = .079), although this difference did not reach statistical significance.

**Conclusions:**

Immunoprofiling luminal vs basal type disease appears to harbor prognostic relevance in mHSPC—this is in keeping with different stages and cancer types. Our preliminary findings warrant further investigation in larger and potentially prospective cohorts. These data may better stratify mHSPC patients and potentially help treatment decision-making, hence avoiding overtreatments. A longer follow-up and larger number of patients are mandatory to validate the role of IHC profiling.

